# The immune phenotype of circulating tumor cells: from escape mechanisms to immunotherapy navigation

**DOI:** 10.1186/s40364-026-01015-w

**Published:** 2026-10-09

**Authors:** Pengfei Zhang, Miao Li, Lixin Li, Jiahao Fu, Qiang Li, Tianxing Liu, Renya Zeng, Yang Zhou, Rudi Mao, Xiaolong Wang, Shuhui Li, Xinyu Zhang, Zhe Yang

**Affiliations:** 1 Tumor Research and Therapy Center, Shandong Provincial Hospital, Cheeloo College of Medicine, Shandong University, Jinan, 250021 Shandong China; 2 Tumor Research and Therapy Center, Shandong Provincial Hospital Affiliated to Shandong First Medical University, Jinan, 250021 Shandong China; 3 Jinan Maternity and Child Care Hospital Affiliated to Shandong First Medical University, Jinan, 250021 China; 4 Department of Urology, Shandong Provincial Hospital Affiliated to Shandong First Medical University, Jinan, China; 5 Cancer Center, Shandong University, Department of Radiation Oncology, Shandong Cancer Hospital and Institute, Shandong First Medical University and Shandong Academy of Medical Sciences, Jinan, Shandong China

**Keywords:** Circulating tumor cells, Immune phenotype, Immune evasion, PD-L1, Immune checkpoint inhibitors, Liquid biopsy, Immunotherapy biomarkers, Metastasis

## Abstract

Circulating tumor cells (CTCs) must survive immune attack in the bloodstream before they can contribute to distant metastasis. Their immune phenotype, including checkpoint ligands, antigen-presentation molecules, and immunomodulatory features, can be assessed through serial blood sampling and may provide information complementary to established immunotherapy biomarkers. This review organizes CTC immune evasion across intravasation, circulatory transit, and colonization, and examines CTC-associated immune interactions while distinguishing direct CTC observations from evidence derived from tumor tissue, cell culture, or animal models. We review technologies for immune phenotyping and the clinical evidence linking CTC immune features to immunotherapy outcomes across cancer types. Current studies support further investigation of longitudinal CTC monitoring, but heterogeneous assays, small or retrospective cohorts, and limited prospective validation prevent routine treatment selection based on these readouts. We also discuss experimental approaches to disrupting CTC immune protection during vascular transit and outline a four-stage pathway from discovery and analytical validation to clinical utility and implementation. CTC immune phenotyping remains a research strategy whose clinical value must be established beyond tissue biomarkers, ctDNA, and standard clinical assessment.

## Introduction

Metastasis remains the principal cause of cancer-related mortality, accounting for approximately 90% of cancer deaths globally [1]. Central to the metastatic process are circulating tumor cells (CTCs)—cancer cells shed from primary or metastatic tumors that enter the bloodstream and possess the potential to colonize distant organs [2, 3]. Since their first description by Thomas Ashworth in 1869, CTCs have evolved from pathological curiosity to clinically validated biomarkers; enumeration via the FDA-cleared CellSearch system has been established as an independent prognostic indicator in metastatic breast, prostate, and colorectal cancers [4, 5].

Immune checkpoint inhibitors (ICIs) have expanded cancer treatment by blocking inhibitory receptor-ligand interactions such as PD-1/PD-L1 and CTLA-4/B7 [6, 7]. Anti-PD-1/PD-L1 therapies can produce durable benefit in several malignancies, including non-small cell lung cancer (NSCLC), melanoma, and urothelial carcinoma [8]. Benefit nevertheless varies across cancer types, treatment regimens, and patient populations, and reliable predictive biomarkers remain a clinical need [9, 10].

Current immunotherapy biomarkers provide useful but incomplete information. Tissue PD-L1 immunohistochemistry (IHC) is affected by spatial and temporal heterogeneity, biopsy sampling, and differences in antibody clones and scoring systems [11, 12]. Tumor mutational burden (TMB) and microsatellite instability (MSI) describe additional aspects of tumor biology, but these measurements do not directly characterize the immune phenotype of intact circulating tumor cells [13]. Serial CTC assessment could therefore be investigated as a complementary source of information about cell-level immune changes during treatment.

Serial blood sampling makes CTC immune phenotyping a candidate approach to monitoring selected tumor-cell states during treatment. CTCs are a selected subpopulation exposed to immune pressure in the tumor microenvironment (TME) and during circulation, so their phenotype need not reproduce that of the primary tumor [14, 15]. Candidate readouts include immune checkpoint ligands such as PD-L1, CD47, B7-H3, and HLA-G, antigen-presentation molecules, and other immunomodulatory features. Studies of the HLA-E–CD94/NKG2A and HLA-C–KIR axes also suggest that innate immune recognition deserves attention alongside T-cell checkpoints [16, 17]. Whether these measurements improve prediction or monitoring of immunotherapy response requires disease-specific clinical validation.

Previous reviews have described CTC biology, immune evasion, and metastatic dissemination [18–22]. Here, we bring these topics together around three questions: how immune pressure changes during dissemination, which CTC-associated immune features can be measured, and what evidence would be needed to use those measurements in immunotherapy studies.

We organize immune-evasion mechanisms by dissemination stage, examine interactions between CTCs and immune cells, and assess candidate biomarkers across cancer types. Direct observations in patient-derived CTCs are distinguished from functional studies in cell culture or animal models and from findings in tumor tissue. These sources answer different questions: detecting a marker on CTCs does not establish its functional role, and demonstrating a mechanism in a model does not establish predictive value in patients. This distinction guides the interpretation of the clinical evidence and the proposed validation roadmap.

By shifting the emphasis from CTC enumeration to CTC immune phenotyping, this review frames circulating tumor cells as dynamic windows into tumor-immune interaction [23–25]. This perspective does not yet establish CTC immune phenotyping as a routine clinical decision tool, but it clarifies the biological rationale, technical requirements, and validation steps needed to test its translational value.

## Stage-resolved mechanisms of CTC immune evasion

**Fig. 1:**
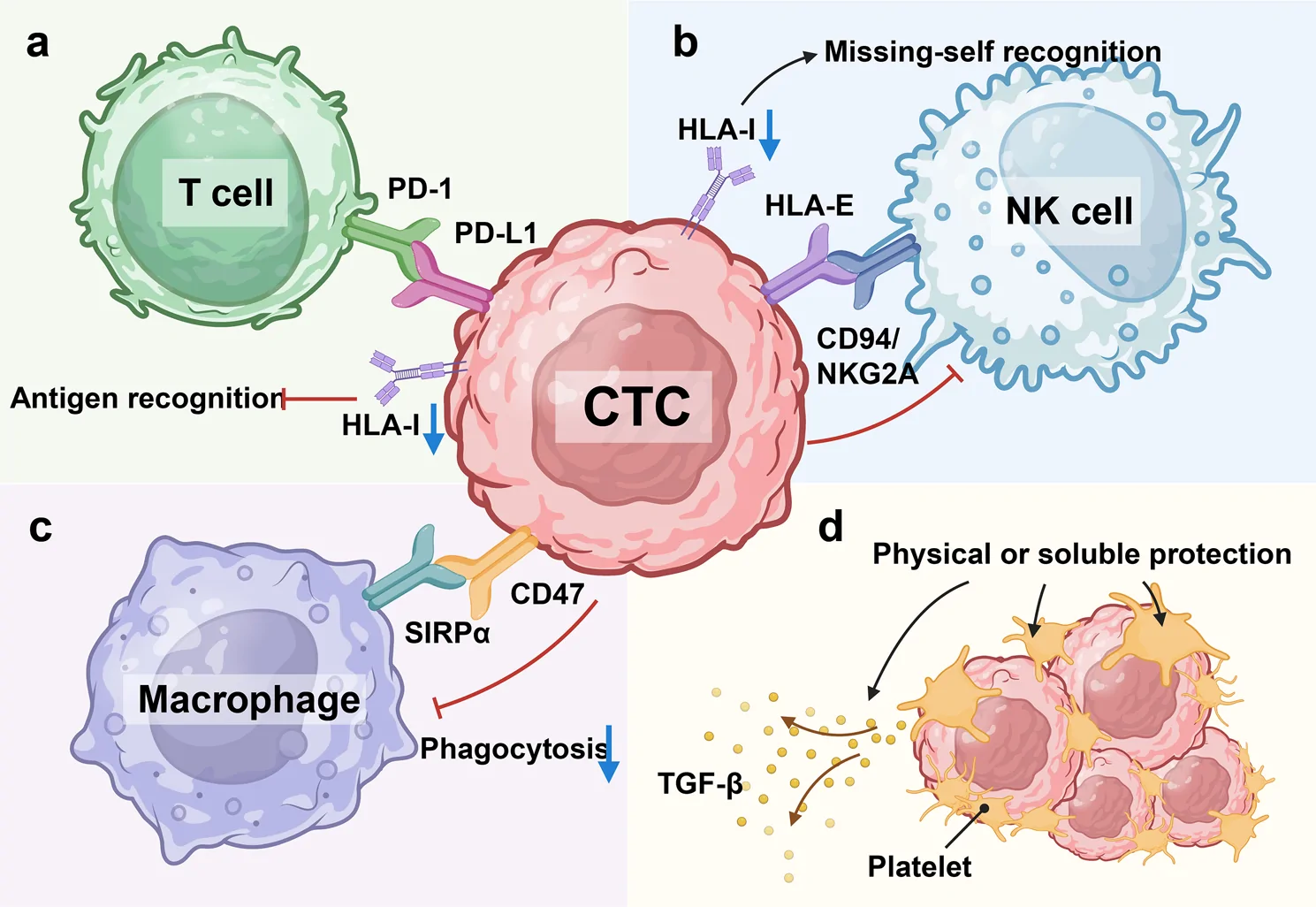
Selected mechanisms of CTC immune evasion. (**a**) PD-L1–PD-1 engagement can inhibit T-cell effector function; reduced HLA-I display can limit antigen recognition. (**b**) HLA-E–CD94/NKG2A signaling can inhibit NK cells, whereas reduced HLA-I display may increase NK-cell missing-self recognition; a and b therefore show two consequences of the same event rather than two independent mechanisms. (**c**) CD47–SIRPα signaling can reduce phagocytosis. (**d**) Platelet coating, clustering and platelet-derived TGF-β provide physical or soluble protection. Paired symbols denote receptor–ligand binding; red blunt-ended lines denote functional inhibition of the effector cell. Evidence differs among pathways and includes experimental-model extrapolation. CTC, circulating tumor cell; NK, natural killer; HLA, human leukocyte antigen; MHC, major histocompatibility complex; PD-1, programmed cell death protein 1; PD-L1, programmed death-ligand 1; SIRPα, signal regulatory protein alpha; TGF-β, transforming growth factor beta

The transit of CTCs from primary tumor to distant metastasis is a multistep process in which immune pressure varies by stage. During shedding and intravasation, survival in the circulation, and extravasation and colonization, tumor cells encounter different immune effector populations and protective interactions [18, 19]. This section follows those stages to distinguish mechanisms operating before vascular entry from those acting in blood or at metastatic sites. Figure 1 focuses on immune-evasion mechanisms during circulatory transit.

### Shedding and intravasation: escape from local immune surveillance

During shedding and intravasation, immune escape can begin before vascular entry. Immune editing within the primary TME may select cells with reduced immunogenicity and increased resistance to immune attack [20]. Comparisons of primary tumors and CTCs, including single-cell analyses, identify differences in antigen-presentation and interferon-response programs consistent with this possibility [21, 22, 26, 27]. Such comparisons do not, by themselves, establish when each phenotype was acquired. They support examining immune evasion as a combination of selection before dissemination and adaptation during transit, rather than assuming that all escape programs arise only in the bloodstream.

A second contributing process is epithelial-mesenchymal plasticity, which links physical dissemination to immune escape. In addition to increasing motility and invasion, EMT-associated programs can reduce antigen presentation, increase checkpoint-ligand expression, and shift CTCs toward hybrid epithelial-mesenchymal states associated with metastatic competence [28–31]. CTC-focused studies also connect mesenchymal features with altered NK-cell sensitivity through N-cadherin-dependent signaling and stromal cGAS-STING-IFN-γ-HLA crosstalk [32, 33]. These findings indicate that plasticity can alter the immune-evasion pathways available to a circulating cell; they do not establish EMT status alone as a marker for selecting immunotherapy.

### Survival during circulatory transit

On entering the bloodstream, CTCs are exposed to multiple sources of stress simultaneously. Beyond hemodynamic shear and anoikis, they encounter the full repertoire of circulating immune effectors: natural killer (NK) cells, monocytes/macrophages, neutrophils, gamma-delta T cells, and complement [34]. The estimated circulating half-life of CTCs is 1–2.4 hours, reflecting strong selective pressure [35]. The strategies CTCs use to survive this interval fall into two groups: cell-intrinsic mechanisms and cell-extrinsic mechanisms that exploit blood components.

#### Cell-intrinsic immune evasion mechanisms

PD-L1 remains the best-studied immune checkpoint ligand on CTCs. Its expression has been reported across cancer types and can differ from matched tissue PD-L1 status, although biological heterogeneity and assay differences both affect this comparison [36–40]. CD47 links CTC phenotyping to macrophage phagocytosis through the SIRP-α axis [41, 42]. HLA-G, HLA-E–CD94/NKG2A, CD155–TIGIT, and vesicle-associated immune modulation represent additional candidate pathways, with varying amounts of direct CTC evidence [43–46]. These pathways should be distinguished from one another and from clinically validated biomarkers.

Loss or remodeling of MHC class I (HLA class I; hereafter MHC-I) can reduce recognition by cytotoxic T cells, whereas loss of inhibitory MHC-I signals can increase susceptibility to NK-cell missing-self recognition. The outcome depends on the balance of activating and inhibitory signals, including retention of non-classical MHC molecules and other NK-inhibitory ligands [47–51]. Survival in suspension adds another layer: anoikis resistance and shear-stress adaptation are not immune escape mechanisms by themselves, but can affect susceptibility to stress and cell death [52–57]. Their contribution should therefore be evaluated together with checkpoint and antigen-presentation changes rather than inferred from survival markers alone.

#### Cell-extrinsic immune evasion mechanisms

Platelet cloaking is the best-characterized extrinsic shield available to CTCs, and it operates through at least three non-exclusive routes. First, activated platelets adhere to the tumor-cell surface soon after intravasation and form a physical coat that reduces the accessibility of activating ligands to NK-cell receptors [58, 59]. Second, platelets transfer platelet-derived MHC class I onto the tumor-cell membrane, producing a pseudo-normal surface phenotype that dampens missing-self recognition; NK-cell reactivity is restored in experimental systems when this transfer is prevented [60]. Third, the platelet releasate, including TGF-β and other soluble mediators, blunts NK-cell effector function, while platelet- and fibrin(ogen)-dependent aggregation impedes NK-cell-mediated elimination of tumor cells in vivo [61]. Platelet-supported CD155–TIGIT signaling provides a further receptor-level route through which the platelet–CTC interface suppresses NK killing [44]. These mechanisms act on recognition, on inhibitory signaling, and on physical accessibility at the same time, which may explain why targeting a single platelet-derived mediator has been less effective than approaches directed at the platelet–CTC interaction itself [62]. Most of this evidence derives from experimental models and ex vivo co-culture rather than from patient-derived CTCs, and the proportion of CTCs carrying a platelet coat in patients has not been systematically quantified.

Neutrophils provide a second protective interface, and the supporting evidence separates into two mechanistically distinct arms that are often conflated. The first is cellular escort. Single-cell profiling of CTC–neutrophil clusters in patients with breast cancer and in mouse models showed that neutrophil-associated CTCs carry transcriptional programs favoring cell-cycle progression, and that such clusters seed metastases more efficiently than CTCs traveling alone [63]. The second arm is NET-mediated. NETs released during systemic inflammation or after surgery sequester CTCs in the hepatic and pulmonary microvasculature and promote adhesion and extravasation [64, 65], and CXCR1/CXCR2-driven NET formation can additionally shield tumor cells from contact-dependent cytotoxicity [66]. NETs have also been shown to awaken dormant disseminated cells through remodeling of laminin [67]. More recent work identifies upstream regulators of these events, including platelet TLR4–ERK5 signaling that couples surgical stress to NET-mediated CTC capture [68] and pericyte-driven formation of CTC–neutrophil clusters in colorectal cancer [69]. Separating escort from entrapment matters therapeutically as well as conceptually: dissolving NETs and preventing cluster formation predict different effects on immune exposure, and an intervention that removes a NET barrier will not necessarily dissociate a neutrophil-containing cluster.

Myeloid-derived suppressor cells (MDSCs) and regulatory T cells (Tregs) have also been associated with higher CTC burden and worse prognosis in several cancers [70–73]. In some settings, CTCs may contribute to this broader immunosuppressive milieu by releasing cytokines, extracellular vesicles, or platelet-activating signals, but the strongest current evidence supports bidirectional association rather than a simple CTC immune suppression pathway [74]. On current evidence, CTCs most likely benefit from and in some settings contribute to a systemic immunosuppressive milieu, but the causal direction remains unresolved.

#### CTC clusters and collective immune protection

Homotypic and heterotypic CTC clusters survive the circulation more efficiently than single cells, and part of this advantage is immunological rather than purely mechanical. Clustering reshapes methylation, stemness, and stress-adaptation programs while the outer cell layers, associated platelets, and recruited neutrophils can reduce exposure of interior cells to shear stress and immune killing [70, 72, 75–78]. The existence of heterotypic CTC clusters therefore strengthens the case for studying CTCs as immune-ecological units rather than isolated tumor cells. It also has a methodological consequence: cluster-focused assays should preserve cell-cell architecture rather than reduce clusters to single-cell marker averages.

### Extravasation and colonization at metastatic sites

At metastatic sites, immune context determines whether disseminated cells are eliminated, held in dormancy, or progress. Pre-metastatic niche formation and dormancy-immune equilibrium provide the conceptual bridge between circulating immune escape and metastatic outgrowth [73, 74, 79, 80]. High-resolution imaging and mechanistic studies show that early disseminated cells interact rapidly with local innate and adaptive immune programs, including oxygen-sensing T-cell states and tumor-cell-intrinsic programs that influence resistance to immune killing [81–83]. These observations support a conservative conclusion: CTC immune phenotype may bias later colonization, but the field still needs direct longitudinal evidence connecting blood-based CTC immune states with metastatic niche outcomes.

Taken together, immune evasion during dissemination involves overlapping mechanisms whose relevance changes with anatomical context. Before vascular entry, immune editing and epithelial-mesenchymal plasticity may influence the cells available for dissemination. During circulation, checkpoint expression, altered antigen presentation, platelet interactions, neutrophil cooperation, and clustering can provide complementary protection. After extravasation, local immune pressure and dormancy programs influence whether disseminated cells are eliminated, contained, or expand. Figure 1 summarizes the circulation-focused component of this framework. The stage-based organization provides a rationale for experimental testing of candidate vulnerabilities, without establishing which intervention will be effective in patients.

## Ctcs as active immunomodulators

CTCs can participate in reciprocal interactions with immune cells during dissemination, and the evidence for this falls into categories that differ substantially in how directly they implicate the circulating cell itself. Evidence for direct contact and heterotypic clusters is therefore considered separately from studies of extracellular vesicles (EVs) and soluble mediators, whose cellular source is often difficult to determine in blood. This distinction is necessary because a systemic immune change associated with CTC burden does not establish that CTCs caused that change: primary and metastatic deposits, stromal compartments and the host immune system all contribute to the same circulating pool, and CTCs represent a numerically tiny fraction of it. The subsections below therefore move from the best-supported interactions, in which the CTC is physically identifiable, toward the least-resolved, in which attribution to CTCs remains inferential.

### CTC-associated extracellular vesicles in immune modulation

Extracellular vesicles (EVs) provide a mechanism by which tumor cells influence immune cells beyond direct contact. Tumor-derived and circulating EVs can carry PD-L1, NKG2D ligands, cytokine-regulatory cargo, and other immune-active molecules [84–90]. In the CTC context, the strongest claim is not that most circulating EVs are CTC-derived, but that CTCs exist in the same vascular compartment where EV-mediated immune modulation occurs. Recent evidence that EV-derived CD45 can coat CTCs and blunt TCR signaling further supports a direct vesicle-CTC immune-evasion interface [46]. The relative contribution of CTCs versus primary or metastatic tumor deposits to the EV pool remains unresolved.

### CTC-associated cytokine and chemokine signaling

Soluble factors provide another possible link between CTCs and systemic immune states. Studies of tumor and stromal biology implicate TGF-β, IL-6/STAT3, VEGF, and chemokine pathways in impaired dendritic-cell maturation, myeloid-cell recruitment, and reduced effector activity [71, 91–94]. These findings establish a rationale for studying the circulating environment, but do not show that CTCs are the principal source of the measured factors. Primary tumors, metastatic deposits, stromal cells, and immune cells may also contribute. Attribution to CTC secretion requires source-resolved experiments; plasma concentrations or correlations with CTC counts alone cannot establish that origin.

### Contact-dependent immune cell reprogramming

Beyond soluble and vesicular signaling, CTCs can also reprogram immune cells through direct physical contact during dissemination. The clearest evidence comes from heterotypic clusters, in which direct apposition to immune cells can be converted from a cytotoxic interface into a pro-metastatic one, and from mesenchymal CTC programs that depend on reciprocal crosstalk with macrophages during colonization [76, 95]. These contact-dependent interactions are mechanistically distinct from EV- or cytokine-mediated effects because they require preserved cell-cell architecture and are therefore best studied in assays that avoid forced dissociation. Maintaining this distinction keeps the focus on measurable CTC-associated immune interactions and avoids over-attributing whole-body immune dysfunction to a rare circulating population.

### CTC-associated systemic immune states and pre-metastatic niches

**Fig. 2:**
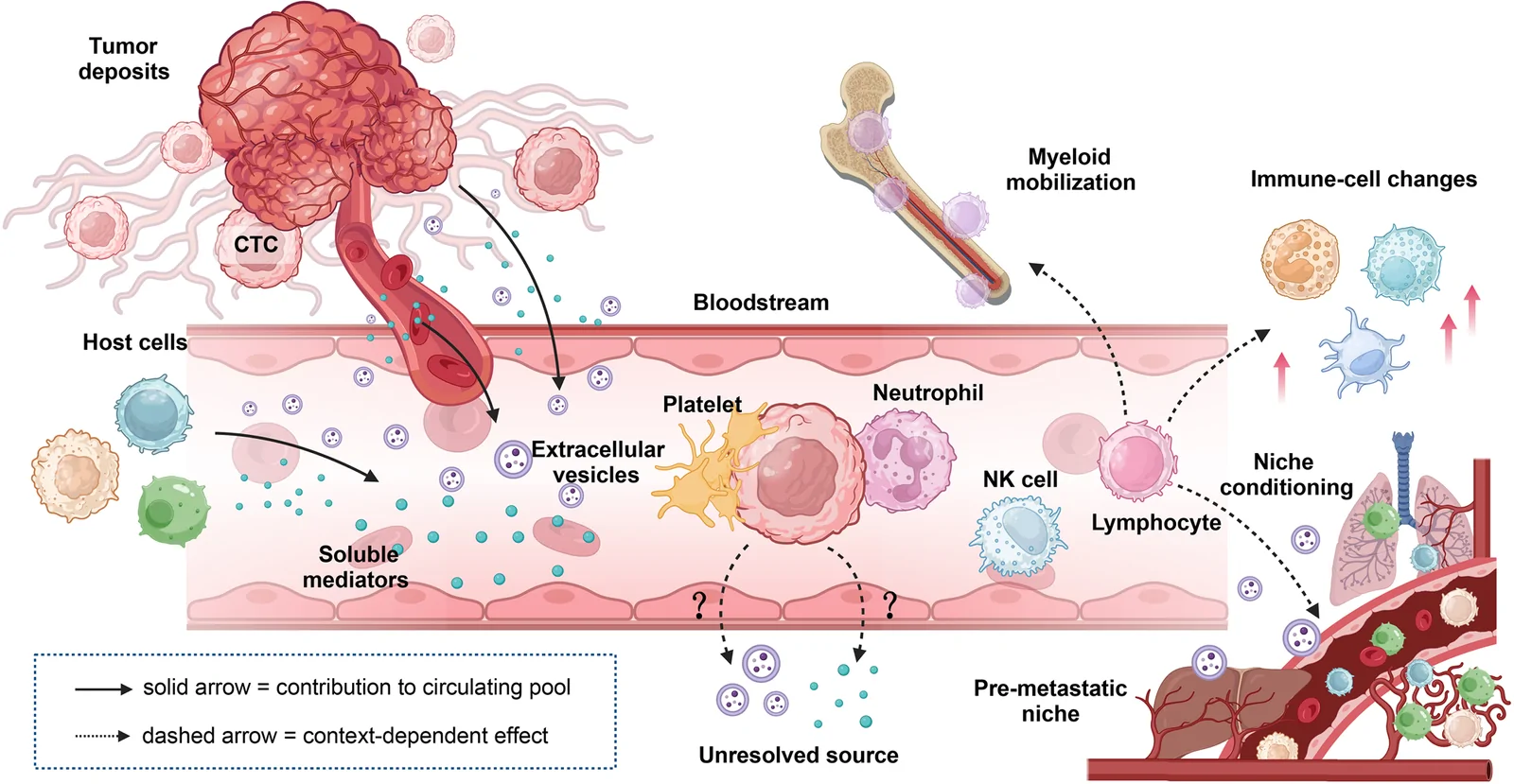
Local CTC interactions and systemic tumor–host signaling. Tumor deposits and host cells contribute extracellular vesicles (EVs) and soluble mediators to blood. CTC–platelet and CTC–neutrophil associations are distinguished from immune-cell changes, myeloid mobilization and niche conditioning. Solid arrows indicate contribution to the circulating pool; dashed arrows indicate context-dependent effects supported by selected experimental models. The contribution specifically attributable to CTCs remains unresolved for many signals, as indicated by the question marks. Plasma measurements and correlations with CTC burden do not establish cellular origin or causality. The diagram does not quantify effect sizes. CTC, circulating tumor cell; EV, extracellular vesicle; NK, natural killer; TGF-β, transforming growth factor beta

Figure 2 distinguishes direct CTC-associated interactions from broader tumor-associated signaling and systemic immune observations. Associations between CTC burden and circulating lymphocyte or NK-cell populations do not resolve whether CTCs drive immune dysfunction or reflect disease burden and the surrounding immune environment [96]. Tumor-derived EVs can contribute to pre-metastatic niche formation in experimental models, but those findings do not establish an equivalent contribution from CTC-derived EVs in patients [97, 98]. CTC profiling may help investigate this relationship when paired with immune-cell measurements and functional studies; it should not be interpreted as a stand-alone measure of CTC-mediated systemic immunosuppression.

## Technological innovations for CTC immune phenotyping

**Fig. 3:**
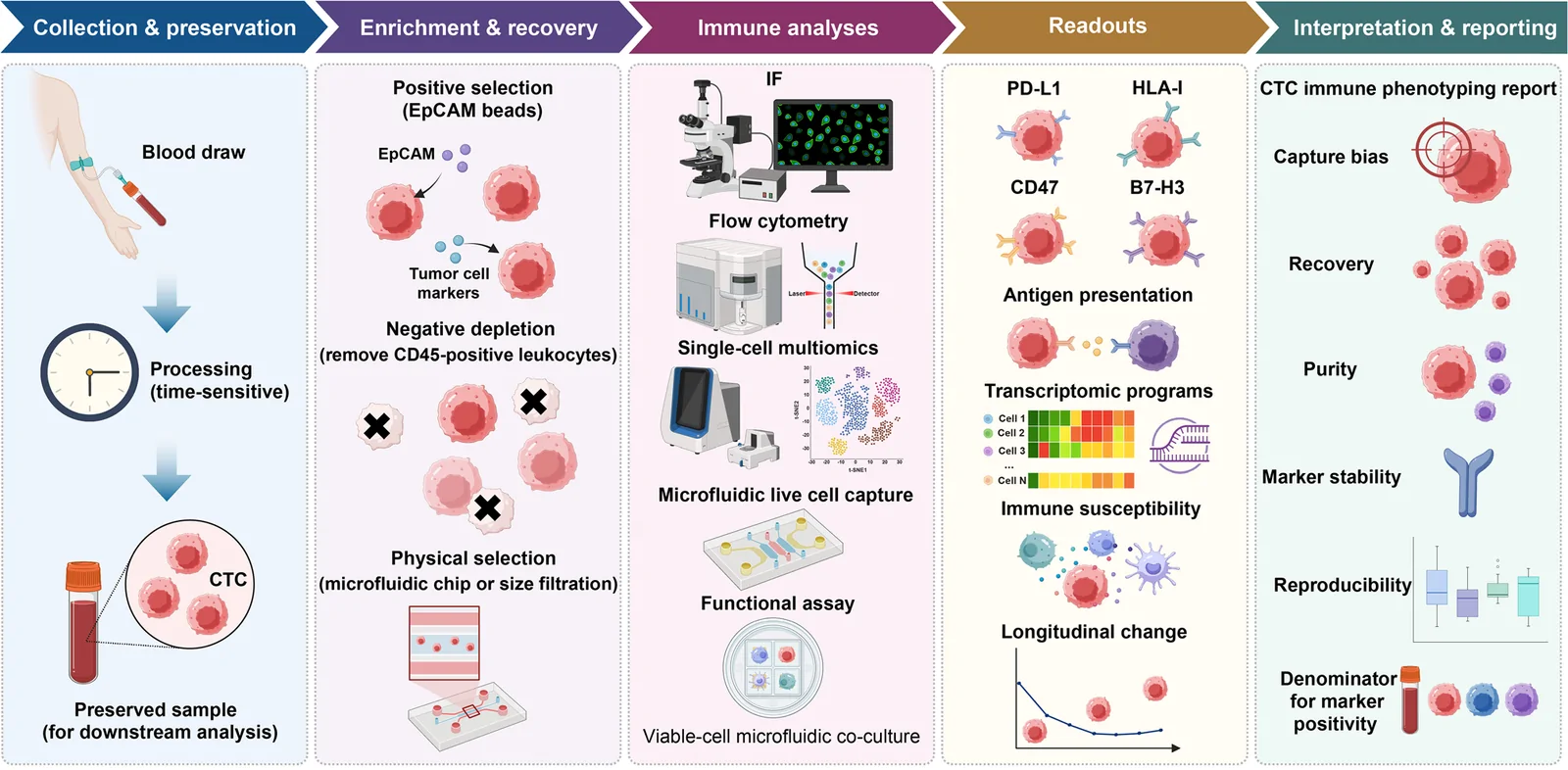
Workflow for CTC immune phenotyping. Collection and preservation precede enrichment, compatible immune analyses and readout interpretation. Positive selection, negative depletion and physical selection are alternative or combinable approaches. Downstream compatibility depends on cell recovery, viability, fixation and assay requirements. Connections represent workflow stages, not one-to-one mappings between capture methods, analytical platforms and readouts. Capture bias, recovery, purity, marker stability, reproducibility and the denominator for marker positivity should be reported. No performance score or universal platform ranking is implied. CTC, circulating tumor cell; IF, immunofluorescence; EpCAM, epithelial cell adhesion molecule; CD45, leukocyte common antigen; HLA, human leukocyte antigen; PD-L1, programmed death-ligand 1; t-SNE, t-distributed stochastic neighbor embedding

CTC immune phenotyping is constrained by low cell abundance, capture bias, marker instability, and limited cross-platform comparability. These constraints are not independent: a capture method chosen to maximize recovery may also select a phenotype, and a fixation protocol chosen to preserve morphology may compromise the epitope to be measured. Assay selection should therefore follow the immune feature of interest—surface proteins, antigen-presentation status, transcriptomic programs, functional immune susceptibility, or longitudinal changes—rather than platform availability [99]. Fig. 3 separates enrichment and recovery from downstream readouts and summarizes practical limitations at each stage. It is a qualitative comparison of assay roles, not a scored performance ranking, and the subsections that follow discuss each class of method in terms of what it can and cannot resolve.

### Antibody-based CTC immune marker detection

Antibody-based platforms remain the practical entry point for CTC immune phenotyping because they can connect established epithelial or lineage capture strategies with clinically interpretable immune markers. Their value is not only analytical sensitivity, but the ability to preserve spatial co-localization between tumor-cell identity and immune-regulatory proteins such as PD-L1, MHC-I/HLA-I, CD47, or immune-lineage exclusion markers. This subsection therefore distinguishes enumeration-centered clinical systems from multiplex imaging approaches that provide richer phenotypic context but require stronger standardization.

#### The CellSearch platform: capabilities and limitations

The CellSearch system provides the most standardized clinical framework for epithelial CTC enumeration, using EpCAM-mediated enrichment followed by cytokeratin, DAPI, and CD45 staining [100]. Its fourth fluorescent channel has been adapted for PD-L1 and other immune markers, making it useful for single-marker clinical studies. However, the same architecture limits immune phenotyping: EpCAM-based capture under-represents mesenchymal or hybrid CTCs, and a single additional channel cannot resolve coordinated changes in PD-L1, MHC-I, CD47, B7–H3, or EMT state. Newer enrichment and nanoparticle-based assays improve detection of phenotypically plastic CTCs, but most remain platform demonstrations rather than clinically standardized immune-phenotyping workflows [101, 102]. Accordingly, CellSearch-derived immune readouts should be interpreted as standardized but incomplete measurements of the circulating compartment.

#### Multiparametric immunofluorescence, flow cytometry and mass cytometry

Multiparametric immunofluorescence, flow cytometry, and mass cytometry extend CTC analysis from single-marker positivity to coordinated immune phenotypes. Immune features should be interpreted at the level actually measured. In the HNSCC cohort discussed below, PD-L1 mRNA was quantified by RT-qPCR in an EpCAM-enriched CTC fraction; this readout is distinct from cell-resolved protein imaging [103]. Flow cytometry and CyTOF can measure larger marker panels, but their value depends on enrichment strategy, event recovery, and the ability to distinguish rare CTCs from leukocyte background [104]. These platforms are complementary readout strategies rather than competing devices: imaging is strongest for morphology-linked marker localization, flow-based methods for surface-marker breadth, and CyTOF for exploratory high-dimensional profiling.

### Single-cell multiomics technologies

Single-cell multiomics is most useful when CTC immune phenotype cannot be reduced to a surface-marker call. CTC transcriptomic studies have revealed patient-level and within-sample heterogeneity in immune checkpoint programs, antigen-presentation machinery, interferon-response states, and metastatic competence, while more recent phenotyping work has emphasized that these states can differ substantially even within the same blood draw [26, 27, 105–109]. Protein-transcriptome approaches such as CITE-seq help bridge the gap between mRNA and surface-protein abundance, and related single-cell platforms provide a framework for linking immune-marker states to functional heterogeneity [110]. The current limitation is translational rather than conceptual: cost, cell recovery, batch effects, and sparse cell numbers still prevent routine clinical implementation.

### Microfluidic technologies and live cell capture

Given that microfluidic platforms maintain cell viability, they also facilitate functional immune assays. Static marker profiling cannot determine whether CTCs resist NK-cell killing, suppress T-cell activation, or evade macrophage phagocytosis. Live-cell capture platforms therefore add functional information by enabling co-culture with immune effector cells and downstream viability or cytotoxicity assays [111, 112]. CTC-derived cultures and ex vivo models extend this logic by allowing repeated testing of antigen-presentation dynamics, IFN-γ responsiveness, antibody-dependent cellular cytotoxicity (ADCC) sensitivity, and drug-immune combinations [113, 114]. These models remain difficult to establish and may select for culture-adapted subpopulations; they remain functional hypothesis-testing systems, not routine clinical assays.

### Technical challenges and standardization needs

Clinical translation will depend less on adding new capture devices than on reducing pre-analytical and analytical variability. Key sources of variation include blood volume, processing delay, enrichment bias, antibody clone, positivity threshold, and whether readouts are scored per cell, per patient, or longitudinally [115, 116]. CTC PD-L1 testing illustrates the problem: studies differ in clone selection, staining workflow, intensity threshold, and whether platelet/leukocyte-associated signal is excluded. Harmonization similar in spirit to tissue PD-L1 comparability efforts may be necessary, but CTC-specific standards must also account for rarity, EMT-associated marker loss, and platform-dependent recovery. Until such standards exist, CTC immune phenotyping should be reported with platform, threshold, denominator, and sampling-time information.

## CTC immune phenotype as an immunotherapy biomarker: cross-cancer evidence

**Fig. 4:**
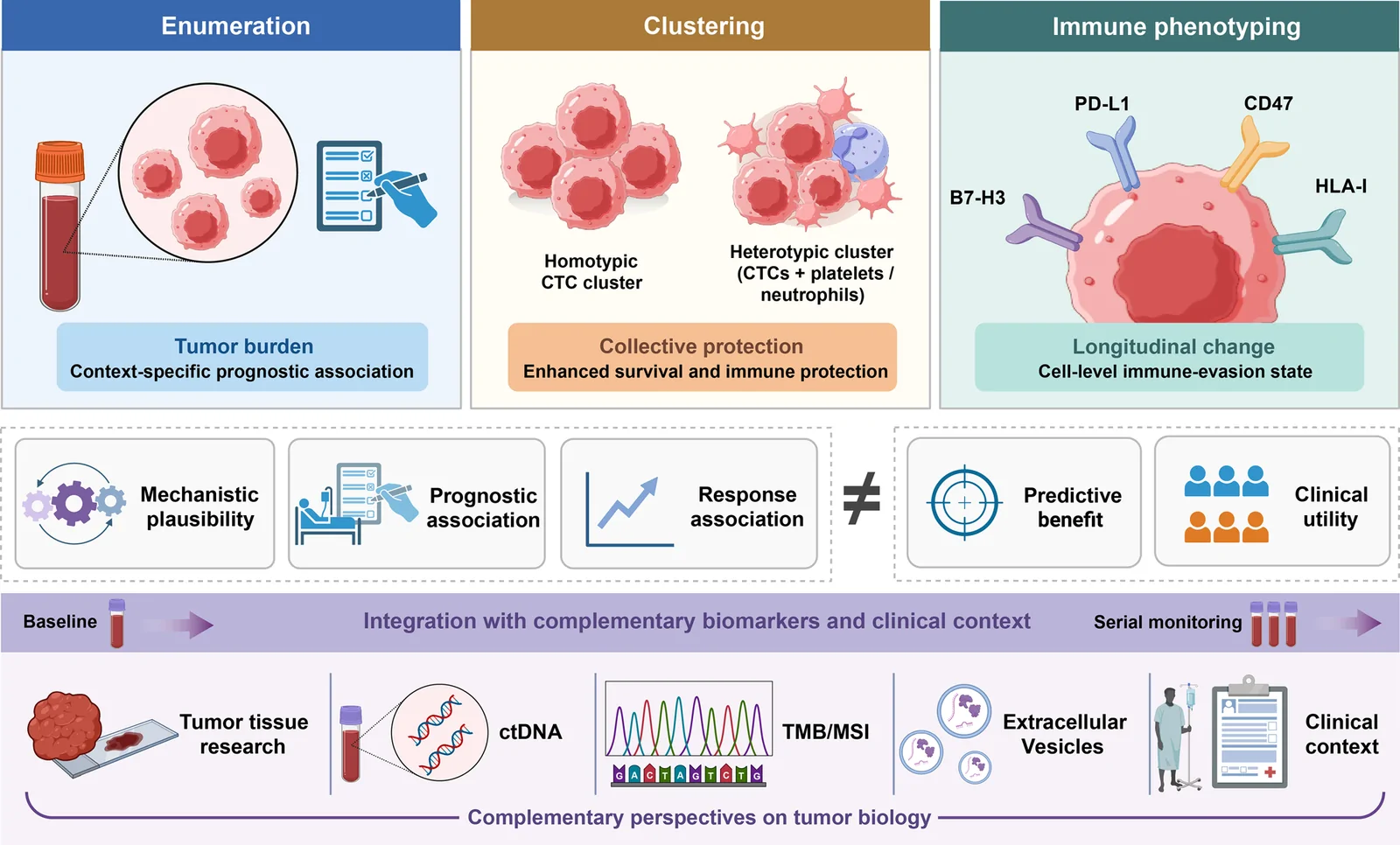
Context and boundaries of candidate CTC readouts. This qualitative summary separates enumeration, clustering and immune phenotyping. Prognostic evidence for CTC count is specific to defined clinical settings. Cluster analyses and immune-phenotype studies differ in assays, populations, thresholds and sampling times. No numerical maturity score or universal evidence ranking is implied. Mechanistic plausibility and prognostic or response associations must be distinguished from evidence that a biomarker predicts treatment benefit or that a biomarker-guided intervention improves patient outcomes; this is the distinction marked in the figure. CTC, circulating tumor cell; ctDNA, circulating tumor DNA; TMB, tumor mutational burden; MSI, microsatellite instability; HLA, human leukocyte antigen; PD-L1, programmed death-ligand 1

This section evaluates evidence linking CTC immune phenotypes to immunotherapy outcomes. Three distinctions organize the assessment, because conflating them is the most common source of overstatement in this literature. Direct CTC studies are distinguished from tissue, ctDNA, EV, and general immunotherapy studies, since a mechanism established in tissue does not transfer automatically to the circulating compartment. Prognostic associations are separated from prediction of treatment benefit, which requires a treatment-by-biomarker interaction and therefore a comparator. And serial monitoring is assessed separately from baseline testing, because the two answer different clinical questions and carry different evidentiary requirements. Figure 4 summarizes the types of evidence available and the questions that remain unresolved, without assigning numerical evidence scores or treatment recommendations.

### CTC PD-L1 expression and ICI efficacy prediction

PD-L1-positive CTCs are best considered a dynamic liquid-biopsy correlate of checkpoint biology rather than a direct substitute for tissue PD-L1 immunohistochemistry. The central question is whether PD-L1 on CTCs adds information about spatial heterogeneity, temporal immune adaptation, or treatment-emergent resistance that static tumor tissue cannot capture. Answering it requires evidence of a specific kind: not merely that CTC and tissue results differ, which sampling and analytical variation alone can produce, but that the CTC measurement tracks outcome after tissue PD-L1 and clinical factors have been accounted for. Few studies have been designed to answer that question directly. The following subsections therefore separate evidence from NSCLC, breast cancer, and other tumor types before comparing CTC PD-L1 with tissue-based assays.

#### Non-small cell lung cancer (NSCLC)

NSCLC remains the main clinical setting for CTC PD-L1 because ICIs are central to treatment and advanced disease often yields detectable CTCs [117]. Early studies linked PD-L1-positive CTCs with poorer outcomes during PD-1 blockade, but platform and threshold heterogeneity limit their use as stand-alone treatment selectors [118]. Limited longitudinal studies suggest that dynamic changes in PD-L1-positive CTCs during treatment may be more informative than a single baseline measurement. The strongest current interpretation is therefore longitudinal risk monitoring, not replacement of tissue PD-L1 or established clinical decision markers.

#### Breast cancer and selected non-NSCLC tumor types

In breast cancer, early work established that PD-L1 can be detected on CTCs, particularly in metastatic disease [37]. Analyses of immunotherapy-treated cohorts suggest possible associations with outcome, but subtype, treatment regimen, and assay platform affect interpretation [119]. In locally advanced head and neck squamous cell carcinoma (HNSCC) treated with induction chemotherapy and concurrent chemoradiotherapy, PD-L1 overexpression in the EpCAM-enriched CTC fraction at the end of treatment was associated with poorer outcomes; this study did not evaluate response to PD-1 blockade [103]. Studies in hepatocellular carcinoma and renal cell carcinoma also support disease-specific investigation rather than cancer-agnostic use of CTC immune phenotypes [120, 121].

#### Cross-cancer comparison of PD-L1-positive CTCs as a predictive biomarker

The studies summarized in Table 1 support investigation of CTC PD-L1 across cancer types, but do not establish a common predictive threshold. Reported positivity and associations with outcome differ by disease, treatment setting, enrichment method, and scoring definition. Serial measurements may capture information missed by a baseline sample, yet their added value requires prospective testing against standard clinical and biomarker assessment. Discordance with tissue PD-L1 may reflect biological differences between compartments as well as sampling and analytical variation; it does not, by itself, demonstrate greater predictive accuracy.

**Table 1:** Selected clinical studies of CTC PD-L1 across cancer types and treatment settings

Cancer context	Assay / setting	Main evidence and interpretation	References
NSCLC	CellSearch or IF; nivolumab	Baseline PD-L1-positive CTCs and serial clearance were linked to outcome; best viewed as longitudinal risk monitoring.	[118]
Advanced solid tumors / NSCLC-focused cohorts	CTC-iChip or multiplex IF; PD-1/PD-L1 blockade	Serial decline or treatment-emergent change in PD-L1-positive CTCs may be more informative than a single baseline measurement, but interpretation remains strongly platform- and cohort-dependent.	[39, 122]
Breast cancer	CTC PD-L1 assays during ICI-based therapy	CTC burden and PD-L1 expression provide complementary but method-dependent liquid-biopsy information.	[119]
HNSCC	RT-qPCR of the EpCAM-enriched CTC fraction during induction chemotherapy and concurrent chemoradiotherapy	Post-treatment PD-L1 overexpression was associated with poorer outcomes in locally advanced HNSCC; this was not an ICI-treated cohort.	[103]
Peripheral-blood PD-L1 cellular assays	Lung cancer blood-based cellular PD-L1 assessment	Peripheral-blood PD-L1 signal was associated with worse survival and may differ from tissue PD-L1.	[123]
HCC / RCC	CTC PD-L1 or longitudinal CTC profiling	CTC immune features were associated with outcomes or treatment-emergent phenotypes, supporting prospective validation.	[120, 121]

#### CTC PD-L1 versus tissue PD-L1: concordance and complementarity

CTC PD-L1 and tissue PD-L1 measurements sample different compartments and may differ in timing and scoring systems. Tissue IHC characterizes sampled tumor tissue, whereas CTC assays examine selected circulating cells; biological and analytical differences both affect their comparison [123]. Combined assessment may provide complementary research information, but prospective evidence is insufficient to use a double-positive or double-negative pattern as a treatment-selection rule. A potential complementary research role is temporal: serial CTC PD-L1 measurements could be tested alongside baseline tissue PD-L1 to determine whether they add information about treatment-associated changes. This possibility does not establish clinical utility.

### Beyond PD-L1: emerging CTC immune biomarkers

While PD-L1 dominates the CTC immune phenotyping literature, limitations of PD-L1 as a predictive biomarker (imperfect sensitivity and specificity, dynamic regulation, platform-dependent detection) have driven exploration of other immune markers on CTCs (Table 2). This exploration is timely, as the immunotherapy drug arsenal targeting multiple immune pathways expands, creating opportunities to match circulating immune phenotypes with more specific therapeutic hypotheses. Where direct CTC-specific cohort data remain limited, Table 2 should therefore be read as a translational and mechanistic map rather than as a list of clinically validated selection markers.

**Table 2:** Emerging CTC immune biomarkers beyond PD-L1

Marker / axis	Core immune function	CTC-relevant interpretation	References
CD47 / SIRP-alpha	Macrophage ‘don’t-eat-me’ signaling	Co-expression with PD-L1 has been described in selected CTC studies; its value for stratifying CD47-axis trials remains to be established.	[124–128]
B7-H3	B7-family immune-inhibitory target	Useful candidate for expanded CTC immune panels, but current support remains early and is not yet based on mature CTC-specific clinical validation.	[129]
MHC-I / MHC-II	Antigen presentation to CD8+ or CD4+ T cells	MHC loss may help explain poor T-cell recognition, whereas MHC-II may indicate antigen-presentation-competent states, but current interpretation remains partly inferential.	[50, 130, 131]
HLA-E / CD94-NKG2A	Innate immune checkpoint suppressing NK/CD8+ activity	CTC-relevant HLA-E-CD94/NKG2A signaling provides a candidate target for functional studies, not a validated treatment-selection marker.	[16, 17, 132]
CD155 / TIGIT	Inhibitory signaling to NK and T cells	Platelet-supported CD155-TIGIT signaling protects CTCs from NK killing and supports non-PD-L1 panels.	[44, 45]
EV-derived CD45	Blunts TCR signaling at the CTC interface	EV-coated CTCs show that immune phenotype can include vesicle-mediated shielding beyond cell-surface ligands.	[46]

Beyond PD-L1, the most clinically plausible CTC immune markers are those that connect a measurable circulating phenotype to a therapeutically actionable immune axis. CD47 is the clearest example because CTC CD47 expression links macrophage phagocytosis, SIRP-alpha signaling, and anti-CD47 strategies [124–128]. B7-H3 remains earlier-stage as a CTC biomarker; current evidence supports including it in multiplex panels, but not yet using it as an independent treatment-selection marker [129, 133]. HLA-I/II status may indicate whether CTCs remain visible to T-cell-mediated therapies, although most supporting evidence still comes from tumor tissue or broader immunotherapy studies rather than direct CTC cohorts [50, 130, 131]. Glycosylation and Siglec-related immune suppression represent an emerging surface-phenotype layer, but should be framed as exploratory until stronger CTC-specific validation becomes available [78, 134–137].

### Longitudinal dynamic monitoring of CTC immune phenotype

Repeated sampling is a practical advantage of CTC analysis, but longitudinal interpretation depends on the treatment setting and the tumor-cell phenotypes recovered by the assay [122, 138]. Changes in PD-L1-positive CTCs can be investigated as candidate pharmacodynamic or risk markers; a decrease does not by itself establish treatment benefit. Experimental studies of clustering, antigen-presentation loss, and CD47 signaling also motivate investigation of resistance-associated CTC states, although they do not validate a CTC-based second-line treatment algorithm [75, 139, 140]. Prospective studies must establish whether these measurements add information beyond total CTC burden, imaging, and established biomarkers.

### Combined analysis of CTC enumeration and immune phenotype

CTC enumeration and immune phenotype should be interpreted together. A high CTC count with PD-L1, CD47, low HLA-I, or cluster enrichment suggests both tumor burden and active immune evasion, whereas immune-marker positivity on rare or declining CTCs may carry different meaning [39, 141]. This combined interpretation also helps distinguish a high-risk biological state from a purely quantitative rise in CTC burden. Cluster fraction may add information because clusters show collective survival and immune-protective features, but this remains a research variable rather than a validated immunotherapy-selection criterion [142, 143].

### Integrating CTC immune information with other liquid biopsy analytes

Integration with other liquid-biopsy analytes is most useful when each analyte answers a distinct question. Circulating tumor DNA (ctDNA) can estimate tumor immunogenicity through blood-based TMB or selected immune-resistance alterations, whereas CTCs report cell-level immune escape and metastatic competence [144]. Exosomal PD-L1 provides a soluble/vesicular measure of systemic PD-L1 exposure [86]. In practice, the value of a combined panel will depend on whether each analyte changes interpretation of response, resistance, or metastatic risk beyond CTC enumeration alone. A practical future panel should therefore be hypothesis-driven and longitudinal, not a maximal list of available analytes.

## Therapeutic strategies targeting CTC immune vulnerabilities

Therapeutic exploitation of CTC immune phenotypes remains less mature than biomarker development, but it provides a useful translational framework. The most plausible strategies do not target CTCs as isolated cells only; they target the protective interfaces that allow CTCs to resist immune clearance, including impaired effector-cell recognition, platelet and NET shielding, macrophage checkpoint signaling, and cluster-mediated survival. This section therefore treats CTC immune vulnerability as a set of targetable states rather than a single therapeutic target (Table 3).

**Table 3:** Therapeutic strategies targeting CTC immune vulnerabilities

Mechanism	Representative strategies	CTC immune rationale	Development status	Candidate biomarkers / key refs
Enhancing immune effector killing	NK-cell activation, checkpoint blockade, and dual CD47/PD-L1 blockade	Increase immune recognition and killing of single CTCs, including partially MHC-I-low or checkpoint-ligand-positive cells	Mostly preclinical or early translational; ICIs are clinically established but CTC-specific effects remain incompletely defined	PD-L1, CD47, NKG2A axis; refs [34, 126, 132]
Disrupting protective shields	Antiplatelet strategies, anti-NET approaches, CD47-SIRP-alpha blockade	Remove platelet cloaking, NET entrapment, or “don’t-eat-me” signaling that protects CTCs from immune clearance	Aspirin and platelet-targeting approaches have clinical precedent; anti-CD47 agents are in development; anti-NET strategies remain early	Platelet cloaking markers, NET markers, CD47; refs [62, 64, 127, 128, 145, 146]
Dissociating CTC clusters	Cardiac glycosides, cadherin-targeting approaches, cluster dissociation plus immune enhancement	Break collective immune privilege and expose individual CTCs to immune surveillance	Preclinical evidence plus early pharmacological proof-of-concept for cluster-size reduction	CTC cluster burden, E-cadherin/N-cadherin, immune phenotypes after dissociation; refs [72, 75, 147]
Combination strategies	ICI plus antiplatelet, anti-NET, anti-CD47, or cluster-disrupting approaches	Combine effector-cell reinvigoration with removal of CTC immune-protective barriers	Preclinical rationale and related early trials; CTC-phenotype-matched trials remain a priority	Matched CTC immune phenotype, PD-L1/CD47/NET/cluster features; refs [64, 72, 118, 124]

### Enhancing immune effector cell killing of CTCs

CTC transit exposes tumor cells to circulating immune effectors and offers an experimental setting in which to test interventions intended to reduce metastatic seeding [14, 34]. NK-directed approaches require attention to both activating signals and inhibitory pathways, including NKG2A [132]. Candidate strategies include antibody-dependent cellular cytotoxicity, engineered effector cells, immune-cell engagers, and checkpoint blockade, but supporting studies often involve tumor models or non-CTC clinical settings [148–150]. Their relevance to CTC clearance must therefore be tested directly. Low MHC-I alone does not establish sensitivity to NK-cell therapy or identify an appropriate engager; target-antigen expression, effector competence, and inhibitory signaling also require evaluation.

### Disrupting CTC immune protective shields

Several CTC immune-evasion mechanisms depend on extrinsic shields rather than fixed tumor-cell-intrinsic programs. Platelets, fibrin, neutrophil extracellular traps, and macrophage checkpoint axes can all create transient protection during vascular transit, making them attractive but biologically delicate targets. Their attraction is that they are, in principle, reversible: unlike a genomic loss of antigen-presentation machinery, a platelet coat or a NET can be removed pharmacologically without altering the tumor cell itself. Their delicacy is that each shield is built from a physiological system with essential functions in hemostasis and host defence. The therapeutic challenge is therefore to weaken these protective interfaces enough to restore immune clearance without causing unacceptable bleeding, inflammatory, or off-tumor immune toxicity.

#### Platelet and NET shield disruption

Platelet cloaking and NETs are attractive because they are extrinsic shields rather than fixed tumor-cell traits. Antiplatelet strategies have epidemiological and experimental support for reducing metastatic risk, but their clinical translation must balance immune exposure of CTCs against bleeding risk [62]. Engineered platelet or platelet-mimetic systems remain proof-of-concept tools for intercepting CTCs or remodeling platelet-mediated niches rather than near-term clinical standards [145]. NET-targeting strategies, including DNase-based degradation or inhibition of NET formation, may similarly expose CTCs to immune attack and reduce metastatic trapping [64, 146]. These approaches are best discussed as shield-disrupting adjuncts, not stand-alone anti-CTC therapies.

#### Anti-CD47/SIRPα blockade

CD47-SIRPα signaling provides a rationale for investigating resistance to macrophage phagocytosis, but evidence from solid-tumor models and non-CTC clinical studies does not establish CTC CD47 as a treatment-selection biomarker [127, 128]. Combined CD47 and PD-L1 blockade has experimental support, motivating studies that link marker co-expression to functional susceptibility [126]. CTC CD47 could be evaluated as a stratification or pharmacodynamic variable in such trials. Expression alone should not be taken to predict benefit, particularly because therapeutic targeting must account for CD47 expression on normal cells.

### Dissociating CTC clusters to expose immune vulnerabilities

CTC clusters provide a second therapeutic target. Cluster dissociation may reduce metastatic efficiency and increase exposure of individual cells to immune killing. Preclinical work linked cardiac glycosides to CTC-cluster disruption, and a recent proof-of-concept trial showed that digoxin can reduce CTC cluster size in metastatic breast cancer [72, 147]. The practical next step is not to claim cluster dissociation as established anti-metastatic therapy, but to test whether cluster reduction changes immune susceptibility and clinical outcome. Such studies should measure both physical cluster disruption and downstream immune recognition, because cluster size alone may not capture the relevant biological effect.

### Combination strategies: ICIs + anti-CTC immune protection mechanisms

Combination strategies should be built around matched vulnerabilities rather than generic “more immunotherapy” logic. For example, PD-L1-positive and CD47-positive CTCs support testing adaptive-plus-innate checkpoint blockade, whereas platelet-coated or NET-associated CTCs support combining ICIs with shield-disrupting approaches [126]. At present, however, most trials do not use CTC immune phenotype as an enrollment or stratification variable. A more realistic near-term endpoint is prospective validation that CTC immune features predict response, resistance, or metastatic progression under defined combination regimens. This would turn CTC phenotyping from a descriptive biomarker into a trial-design tool for matching immune escape mechanisms to combination therapy.

## Challenges, future directions, and clinical translation roadmap

Clinical translation requires reproducible measurement, interpretable biological associations, and evidence that CTC immune information improves decisions beyond established biomarkers. The literature considered here has several limitations: cohorts are often small or retrospective; enrichment methods, marker thresholds, denominators, and sampling times differ; and mechanistic support frequently comes from tissue or experimental models rather than paired functional studies of patient-derived CTCs. These differences limit cross-study comparison and separation of predictive effects from general associations with tumor burden or prognosis. In addition, this narrative synthesis does not provide a pooled effect estimate or a formal quantitative ranking of biomarker utility. The priorities below address these limitations.

### Core unresolved questions

Representativeness is a central biological limitation. CTCs are a selected and rare fraction of the tumor population, and their immune phenotype may not mirror the primary tumor or metastatic lesions [151]. Comparative studies of primary tumors, metastatic sites, and matched CTCs should establish which features are shared, which are circulation-associated, and which add information beyond tissue biomarkers [152]. Failure to detect a CTC subset must also be distinguished from true biological absence, particularly when the enrichment method depends on markers altered during dissemination.

Single time-point testing versus longitudinal monitoring. Serial sampling is the practical advantage that distinguishes CTC analysis from tissue biopsy, yet the timing, frequency and interpretation of repeated immune phenotyping remain undefined [153]. Several questions are unresolved: how soon after treatment initiation a change in checkpoint-ligand expression becomes detectable; whether an early on-treatment change carries more information than the baseline phenotype; whether marker changes should be expressed as a proportion of recovered CTCs or as an absolute count of marker-positive cells, given that both numerator and denominator move during therapy; and what magnitude of change exceeds assay and biological variability. Prospective studies with prespecified sampling windows, and with technical replicates sufficient to estimate measurement variability, are needed before serial phenotyping can be interpreted at the level of the individual patient.

Universal versus disease-specific marker panels. Core markers such as PD-L1, MHC-I, and CD47 may be broadly informative because the pathways they report operate across tumor types, but tumor lineage, treatment history and metastatic site are likely to determine which immune features carry information in a given setting. The relevant axis in a tumor treated with PD-1 blockade is not necessarily the relevant axis in one treated with an antibody–drug conjugate or a cellular therapy, and markers reflecting innate recognition may matter more where adaptive recognition is already lost. A practical strategy is therefore a consensus backbone panel, measured identically across studies to permit pooling, combined with disease- or therapy-specific add-on markers. This preserves comparability without forcing biologically distinct settings into a single panel, and it allows the backbone to be revised as evidence accumulates rather than fixed prematurely.

### Technical advancement priorities

High-dimensional and standardized phenotyping platforms. Integrating microfluidic CTC enrichment with multiplex antibody imaging, imaging mass cytometry, or single-cell proteomic readouts could enable simultaneous assessment of 20–40 immune and lineage markers on the same rare cell [154]. The value of such panels, however, depends less on marker number than on comparability: a 30-marker panel that cannot be reproduced in a second laboratory contributes less to translation than a five-marker panel with defined thresholds and known inter-laboratory variation. Priorities are therefore harmonized capture chemistry, shared staining and imaging protocols, common definitions of positivity and of the denominator against which it is expressed, and quality-control metrics—spike-in recovery, leukocyte carry-over, and signal stability across processing delays—reported alongside every dataset. Multicenter ring studies using shared reference materials would be the most direct way to establish whether a given panel is transferable.

Computational recognition and integrative modeling. AI-assisted image analysis may improve the detection of rare CTC subsets and reduce observer-dependent variability in cell classification, which is a documented source of disagreement even among experienced readers [155, 156]. Beyond detection, computational models should integrate CTC immune phenotypes with tissue pathology, ctDNA, exosomal markers, treatment history, and clinical outcomes, and test whether multi-modal liquid biopsy panels outperform single-analyte approaches [157]. Two requirements apply to this work. Models must be trained and validated on separate cohorts, because the number of candidate features in high-dimensional CTC data greatly exceeds the number of patients in most studies and overfitting is the expected failure mode. And model outputs must remain interpretable at the level of the underlying immune mechanism if they are to generate testable hypotheses rather than opaque risk scores.

Emerging in vivo and continuous-monitoring technologies. Photoacoustic flow cytometry, intravascular capture devices, and implantable or wearable sensing concepts may eventually extend CTC monitoring beyond intermittent blood draws [158, 159]. In vivo approaches interrogate a far larger blood volume than a standard draw, which addresses one of the central statistical limitations of CTC analysis: at low CTC concentrations a 7.5 mL sample may contain no tumor cells even when the circulating burden is substantial. The current constraint is that these platforms mostly detect and enumerate rather than phenotype, so they do not yet deliver the immune-marker information discussed in this review. They remain early-stage, but they highlight a long-term direction: moving from static snapshots toward dynamic surveillance of tumor-immune evolution.

### Clinical translation advancement roadmap

**Fig. 5:**
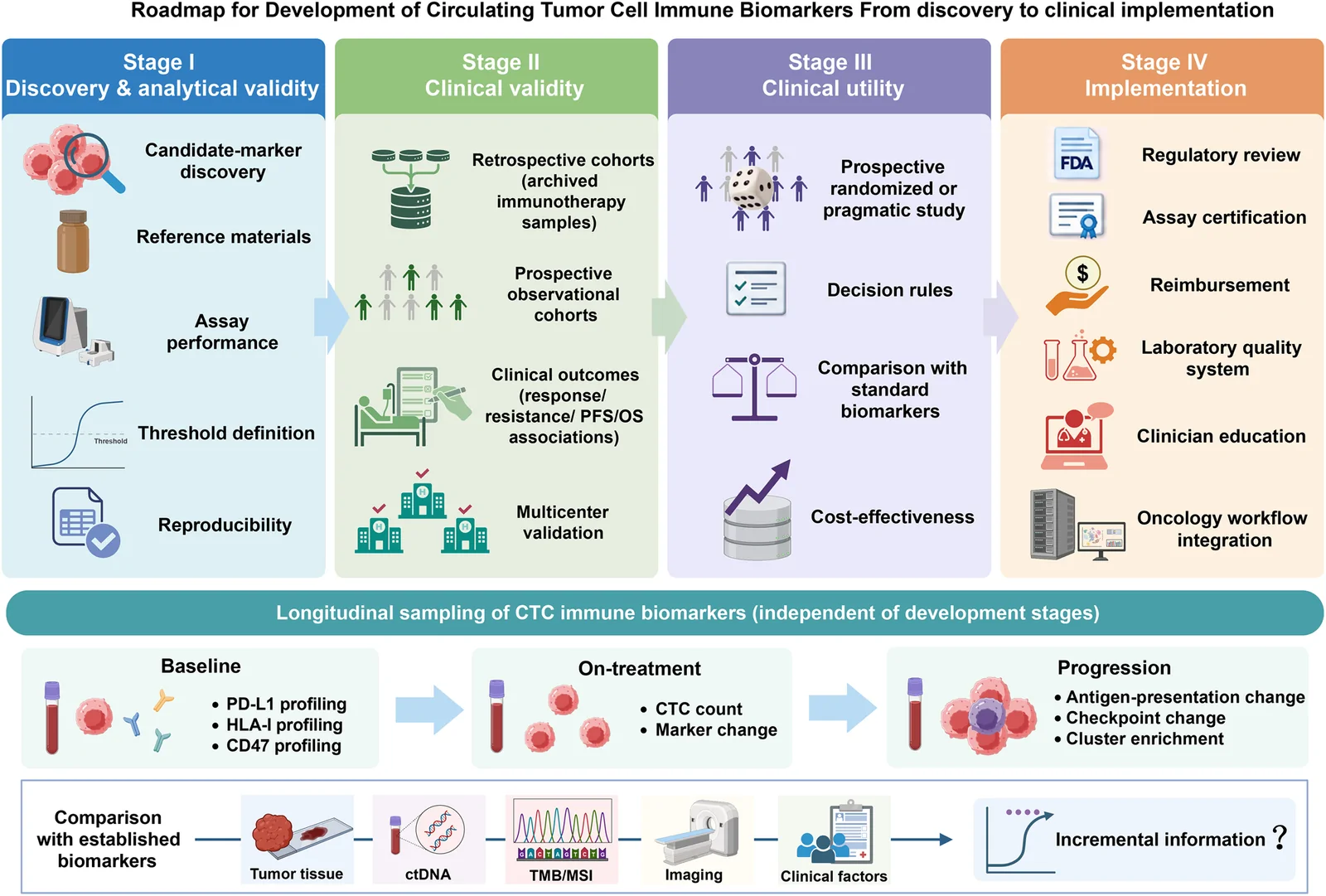
Development pathway for CTC immune biomarkers. Four stages address discovery and analytical validation, clinical validity, clinical utility and implementation. The longitudinal scenario illustrates baseline, on-treatment and progression sampling in prospective studies; these are sampling windows, not treatment triggers or additional development stages. Assays, sampling windows and endpoints should be prespecified. Incremental information should be assessed beyond tissue biomarkers, circulating tumor DNA (ctDNA) and clinical factors. The framework does not recommend changing treatment on the basis of a CTC phenotype. CTC, circulating tumor cell; ctDNA, circulating tumor DNA; TMB, tumor mutational burden; MSI, microsatellite instability; PFS, progression-free survival; OS, overall survival; HLA, human leukocyte antigen; PD-L1, programmed death-ligand 1

As with other liquid-biopsy technologies, moving CTC immune phenotyping from research assays to clinically useful tests will require stepwise validation rather than a single confirmatory study. Each stage closes a different gap: showing that an assay measures the intended immune feature reproducibly, that the feature associates with clinically relevant outcomes, and finally that acting on it improves treatment selection, monitoring, or trial design. The roadmap below therefore organizes this progression into four sequential stages (Fig. 5), each with distinct deliverables and validation priorities.

Stage I: Discovery and analytical validation. The objective of this stage is to demonstrate that an assay measures the intended immune feature reproducibly, before any outcome association is examined. Candidate markers should be selected on the basis of a defined immune mechanism and a plausible therapeutic axis rather than on the availability of an antibody. Assay performance should then be quantified in terms of analytical sensitivity and specificity, limit of detection for rare events, and recovery of spiked tumor cells across the range of CTC counts encountered clinically. Because CTC immune readouts are strongly affected by pre-analytical handling, the blood-collection tube, processing delay, fixation and enrichment chemistry should be fixed and reported as part of the assay definition rather than treated as laboratory preference. Priority needs at this stage include reference materials such as characterized cell lines spiked into normal blood at defined concentrations, standardized reporting templates, inter-laboratory reproducibility studies, and explicit definitions of positivity that specify both the intensity threshold and the denominator—per cell, per CTC-positive patient, or per millilitre of blood—against which positivity is expressed.

Stage II: Clinical validation. Once analytical performance is established, the question becomes whether the measured phenotype tracks clinically meaningful outcomes. Retrospective analyses of archived samples from completed immunotherapy trials offer the fastest route to an initial signal, because treatment, timing and endpoints are already standardized, but they are limited by sample availability, storage-related marker degradation, and the selection inherent in banked cohorts. Prospective observational studies with prespecified sampling windows are therefore required to confirm any retrospective association. Analyses should distinguish prognostic associations, which describe outcome regardless of treatment, from predictive associations, which require an interaction between the biomarker and the treatment received and therefore a comparator arm. Endpoints should be harmonized across contributing centers, and effect estimates should be adjusted for CTC burden, disease setting and line of therapy, so that an apparent immune-phenotype effect is not simply a restatement of tumor burden. Priority needs include access to well-annotated cohorts, prespecified statistical analysis plans, and multicenter validation using a locked assay.

Stage III: Clinical utility demonstration. Clinical validity does not establish that measuring a biomarker helps patients. Demonstrating utility requires a comparison in which the CTC immune readout changes management: randomized biomarker-strategy designs, enrichment designs that restrict enrolment to a defined CTC immune phenotype, or pragmatic studies embedded in routine care. The decision rule must be fixed in advance—which phenotype triggers which action, at which time point—because a rule derived after outcomes are known cannot be evaluated in the same dataset. These studies should also quantify the incremental contribution of the CTC readout over the information already available from tissue biomarkers, ctDNA, imaging and clinical factors, since a marker that is merely correlated with existing tests adds cost without adding decisions. Priority needs include predefined decision rules, formal assessment of incremental value, cost-effectiveness analysis, and head-to-head comparison with established tissue and liquid biopsy assays.

Stage IV: Implementation and regulatory adoption. If clinical utility is demonstrated, the remaining obstacles are operational rather than scientific. Deployment requires regulatory review of the assay in its locked configuration, laboratory quality systems capable of supporting a rare-event test, reimbursement pathways, and turnaround times compatible with treatment decisions—a result that arrives after the next cycle has started cannot influence care. Reporting formats must convey not only the marker call but also the denominator, the number of CTCs assessed, and the limits of interpretation when few or no CTCs are recovered, so that a negative result is not read as evidence of a low immune-evasion burden. Priority needs include assay certification, external quality assessment schemes, clinician education on interpretation, turnaround-time benchmarks, and evidence packages structured for guideline consideration.

### A conceptual framework for testing CTC immune phenotyping in precision immunotherapy

The sampling framework in Figure 5 links baseline, on-treatment, and progression measurements to research questions. Each use requires a predefined assay, sampling schedule, endpoint, and analysis plan, with CTC findings evaluated alongside tissue PD-L1, TMB/MSI where relevant, ctDNA, and clinical context. The examples below are hypotheses for prospective studies; they do not specify when to start, continue, escalate, or switch treatment.

At baseline, studies could test whether PD-L1 expression together with antigen-presentation status defines reproducible CTC subgroups that add information beyond established biomarkers. A PD-L1-positive, MHC-I-intact phenotype does not itself demonstrate ICI sensitivity. Low MHC-I provides a rationale for examining NK-cell susceptibility, but functional studies must also assess inhibitory ligands, activating signals, and effector-cell competence. CD47 expression can similarly motivate phagocytosis assays and exploratory stratification. A marker-negative result may reflect limited assay coverage or cell recovery and should not be interpreted as a low immune-evasion burden or favorable prognosis.

During treatment, prospective studies could test whether changes in absolute CTC counts, the proportion expressing PD-L1, and other prespecified immune features are associated with subsequent response or progression. Total cell recovery and sampling variability should be considered when interpreting apparent marker changes. Declining, persistent, or newly detected immune-marker-positive CTCs are research observations to compare with imaging and clinical outcomes, not independent grounds for continuing or escalating therapy. Newly detected CD47 or B7–H3 expression may warrant mechanistic investigation within the study protocol.

At progression, paired samples could test whether changes in antigen presentation, CD47 expression, other checkpoint pathways, or clustering accompany acquired resistance. These associations would require confirmation with matched tissue, complementary blood measurements, or functional assays as appropriate. Cluster enrichment, for example, could motivate experiments assessing both cluster disruption and immune susceptibility. Such studies can inform trial hypotheses, but a newly detected CTC feature alone cannot establish the cause of resistance or select the next treatment.

## Conclusion

CTC immune phenotyping extends enumeration by examining how circulating cells interact with immune surveillance. Serial sampling can identify changes in checkpoint expression, antigen-presentation molecules, and cell-associated protective features, but the interpretation of those changes depends on tumor type, treatment, and assay recovery. Organizing the evidence by dissemination stage helps distinguish mechanisms operating in blood from those studied in primary tumors or metastatic sites.

Current evidence supports prospective investigation rather than routine immunotherapy selection based on CTC immune readouts. PD-L1 has received the most clinical attention, while antigen-presentation status, CD47, NK-inhibitory pathways, and clustering remain candidate biological or monitoring variables. Direct interactions involving CTCs should be distinguished from broader tumor-associated immune modulation, for which the contribution of CTCs is often unresolved. Small cohorts, assay heterogeneity, and reliance on model-based mechanisms limit the conclusions that can be drawn across cancers.

The next step is to establish whether reproducible CTC immune measurements improve prediction, monitoring, or trial stratification beyond tissue biomarkers, ctDNA, total CTC burden, and standard clinical assessment. This requires defined thresholds and denominators, longitudinal multicenter studies, and prospective demonstration of clinical utility. Until those conditions are met, the proposed figures and framework should guide research questions rather than individual treatment decisions.

## Data Availability

No datasets were generated or analysed during the current study.
